# Supplementary material for: Exposure to the Danish Mandatory Vitamin D Fortification Policy in Prenatal Life and the Risk of Developing Coeliac Disease—The Importance of Season: A Semi Ecological Study
Source: Nutrients. 2020 Apr 27;12(5):1243. doi: 10.3390/nu12051243 (PMC7281975; doi:10.3390/nu12051243)
Supplement: Supplementary file 1 [file nutrients-12-01243-s001.zip › Table S1 and S2.docx]

**Table S1.** Coeliac disease (altered definition) among those prenatally exposed to extra vitamin D from the margarine fortification policy compared to those unexposed.

|  |  | Odds Ratio^1^ | 95% CI^2^ | p-value |
| --- | --- | --- | --- | --- |
| Vitamin D policy^3^ |  |  |  | 0.34 |
|  | Not exposed (ref) | 1 |  |  |
|  | exposed | 0.94 | 0.83;1.07 |  |
| Gender |  |  |  | <0.001 |
|  | Women (ref) | 1 |  |  |
|  | Men | 0.67 | 0.59;0.76 |  |
| Season of birth^4^ |  |  |  |  |
|  | Nov-Jan (winter)(ref) | 1 |  |  |
|  | Feb-Apr (spring) | 0.96 | 0.80;1.16 | 0.68 |
|  | May – Jul (summer) | 1.11 | 0.93;1.32 | 0.27 |
|  | Aug-Oct (autumn) | 0.99 | 0.82;1.19 | 0.88 |

^1^ Likelihood ratio test for interaction between policy and season of birth p=0.72. ^2^ CI = Confidence interval. ^3^Adjusted for gender and season of birth. ^4^Adjusted for gender and policy

**Table S2.** Coeliac disease among those prenatally exposed to extra vitamin D from the margarine fortification policy compared to those unexposed using altered definition of season

|  |  | Odds Ratio^1^ | 95% CI^2^ | p-value |
| --- | --- | --- | --- | --- |
| Gender |  |  |  | >0.001 |
|  | Women (ref) | 1 |  |  |
|  | Men | 0.54 | 0.44;0.68 |  |
| Season of birth^3^ |  |  |  |  |
|  | Dec-Feb (winter)(ref) | 1 |  |  |
|  | Mar-May (spring) | 1.41 | 1.02;1.95 | 0.04 |
|  | Jun – Aug (summer) | 1.66 | 1.21;2.28 | <0.01 |
|  | Sept-Nov (autumn) | 1.31 | 0.94;1.84 | 0.11 |

^1^ Likelihood ratio test for interaction between policy and season of birth p=0.216. ^2^ CI = Confidence interval. ^3^Adjusted for gender
